# STGLR: A Spacecraft Anomaly Detection Method Based on Spatio-Temporal Graph Learning

**DOI:** 10.3390/s25020310

**Published:** 2025-01-07

**Authors:** Yi Lai, Ye Zhu, Li Li, Qing Lan, Yizheng Zuo

**Affiliations:** 1 Innovation Academy for Microsatellites of Chinese Academy of Sciences, Shanghai 201304, China; 2 University of Chinese Academy of Sciences, Beijing 101408, China; 3 Key Lab for Satellite Digitalization Technology of Chinese Academy of Sciences, Shanghai 200031, China

**Keywords:** anomaly detection, spacecraft telemetry data, dynamic graph learning, GraphSAGE, variational auto-encoder

## Abstract

Anomalies frequently occur during the operation of spacecraft in orbit, and studying anomaly detection methods is crucial to ensure the normal operation of spacecraft. Due to the complexity of spacecraft structures, telemetry data possess characteristics such as high dimensionality, complexity, and large scale. Existing methods frequently ignore or fail to explicitly extract the correlation between variables, and due to the lack of prior knowledge, it is difficult to obtain the initial relationship of variables. To address these issues, this paper proposes a new method, namely spatio-temporal graph learning reconstruction (STGLR), for spacecraft anomaly detection. STGLR employs a dynamic graph learning module to infer the initial relationships among telemetry variables. It then constructs a spatio-temporal feature extraction module to capture complex spatio-temporal dependencies among variables, leveraging a graph sample and aggregation network to learn embedded features and incorporating an attention mechanism to adaptively select salient features. Finally, a reconstruction module is used to learn the latent representations of features, capturing the normal patterns in telemetry data and achieving anomaly detection. To validate the effectiveness of the proposed method, experiments were conducted on two public spacecraft datasets, and the results demonstrate that the performance of the STGLR method surpasses existing anomaly detection methods, with an average F1 score exceeding 0.97.

## 1. Introduction

With the continuous advancement of space technology, the number of spacecraft has rapidly increased, and their systems have become increasingly complex. The harsh space environment often exposes spacecraft to unpredictable anomalies and malfunctions during exploration missions [1]. To ensure the proper functioning of spacecraft, it is crucial to develop effective anomaly detection methods. Spacecraft health monitoring is typically achieved through the real-time collection of relevant signals using onboard sensors, followed by their transmission to ground control stations for telemetry data acquisition and analysis. Telemetry data provide critical insights into the operational status and performance of the spacecraft, serving as the primary means for determining whether the spacecraft is functioning normally or exhibiting abnormal behavior.

**Figure 1:**
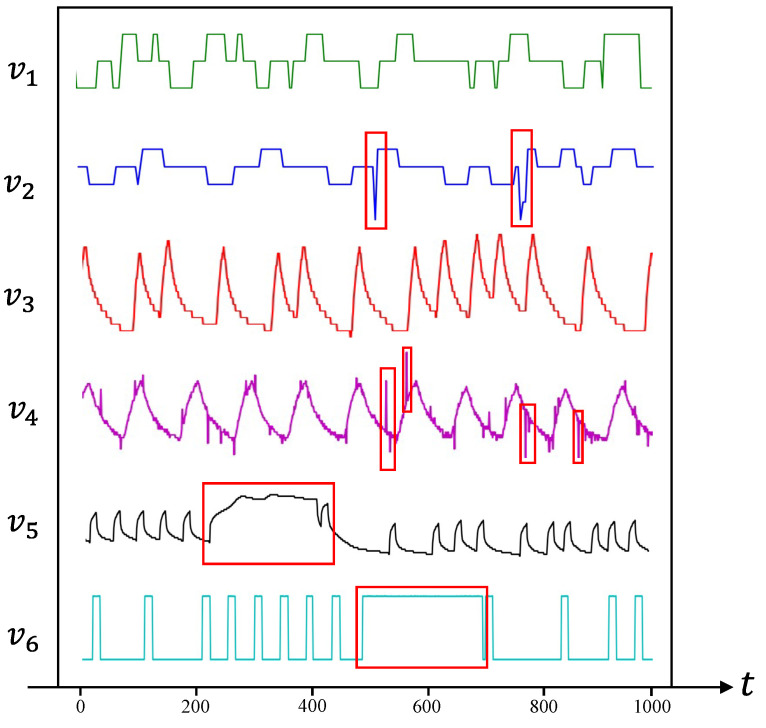
Variables in the SMAP dataset with typical point anomalies in the red boxes in v2 and v4, and collective anomalies in v5 and v6.

Due to the complexity of spacecraft systems and the large number of sensors involved, telemetry data typically exhibit characteristics such as high dimensionality, large scale, and imbalance, making them complex multi-variable time series data to some extent. Traditional anomaly detection methods, including threshold-based [2] and model-based [3] approaches, heavily rely on expert knowledge and are ineffective at identifying unknown anomalies, thereby posing significant challenges for anomaly detection [4]. As a result, more advanced data-driven methods have emerged as a prominent research direction [5]. Numerous data-driven algorithms have been proposed, exhibiting excellent performance in various domains. Early approaches include statistical models such as Autoregressive Integrated Moving Average (ARIMA) [6] and Gaussian Mixture [7]. In addition, various classical algorithms based on distance, clustering, and similarity metrics have been developed [8,9,10], but these methods have limited effectiveness for non-linear, high-dimensional telemetry data. Currently, researchers mostly employ advanced deep learning methods [11] to model time series, learning the normal characteristics of time series through prediction [12,13,14,15] or reconstruction [16,17,18], and thus detecting anomalies. However, when dealing with non-Euclidean domain data, such as telemetry data, most methods tend to overlook or fail to explicitly establish correlations between variables [19]. Our research primarily focuses on analyzing spacecraft telemetry data, with an emphasis on the overall status of the spacecraft system rather than channel-level anomalies. Overall spacecraft anomalies typically manifest as simultaneous anomalies in multiple variables in the telemetry data. Therefore, methods for detecting anomalies in individual variables are unable to capture the changing correlations between different variables. Modeling the temporal and spatial correlations of time series is advantageous for improving anomaly detection effectiveness [20]. Anomalies in spacecraft telemetry data often involve multiple variables, as shown in Figure 1. Currently, the most effective approach to this problem is based on graph neural networks (GNNs) [21,22,23]. Atkinson et al. [24] introduced a GNN-based method for time series anomaly detection, utilizing graph attention networks ( GANs) to learn relationships between sensors and detect data deviating from normal patterns. MTAD-GAN [25] employs GANs to learn spatio-temporal features of multivariate time series and combines prediction and reconstruction models, achieving excellent anomaly detection performance. Thanks to the powerful feature extraction capabilities of GNNs for graph-structured data, GNNs are capable of accurately modeling relationships among multiple variables, demonstrating excellent performance.

The effective use of graph neural networks relies heavily on the accurate construction of the graph structure, as the initial relationships between nodes significantly impact the feature extraction capability of the GNN model. However, for telemetry data from spacecraft, it is challenging to obtain the initial representation graph of relationships. Therefore, how to accurately construct a graph for telemetry data is a major challenge. Most methods typically use predefined or learned fixed graphs to initialize variable relationships. For example, GDN [26] learns sensor relationships by computing similarities between embedding vectors and captures variable correlations using the attention mechanism-based GAN. However, these methods struggle to accurately and comprehensively characterize the relationships between variables within the data. Currently, the mainstream approach is to use dynamic graph learning methods [27]. DyGraphAD [28], proposed by Katrina Chen et al., uses the dynamic time warping (DTW) strategy to address the time offset issue present in Euclidean distance and performs anomaly detection based on deviations from normal to abnormal states using time series. GRELEN [29] proposes a graph relation learning network that detects anomalies by identifying relationship changes between sensors and captures correlations between sensors using a random graph relation learning strategy. SDGL [30] models unknown patterns and infers static and dynamic graphs to capture long-term fixed patterns and short-term dynamic change relationships between variables, enabling time series prediction. However, these methods still present several challenges. Firstly, most approaches construct undirected graphs, whereas real-world scenarios often involve directed relationships between variables. Secondly, these methods require substantial computational resources, resulting in high time and space complexity. When handling high-dimensional and complex telemetry data, it is essential to construct a graph that accurately captures the intricate relationships between variables and to employ appropriate methods for updating node features. Moreover, although an increasing number of approaches consider variable relationships, they frequently neglect the unequal importance and varying impact of different variables on anomaly detection.

To address the aforementioned challenges, we propose an unsupervised anomaly detection framework for spacecraft telemetry data, named space–time graph learning and reconstruction (STGLR). The framework incorporates a dynamic graph learning module to capture the complex relationships among multiple variables over an extended period. We employ the graph sample and aggregation (GraphSAGE) network, which leverages neighbor node sampling and feature aggregation, to model spatial dependencies and accurately capture inter-variable relationships. Subsequently, the embedded features are fed into a gated recurrent unit (GRU) [31] model integrated with an attention mechanism. This model can adaptively select the most relevant input features and learn the long-term temporal dependencies of the telemetry data. Finally, the data are passed through a reconstruction module to learn a latent feature representation of the telemetry data, facilitating effective anomaly detection. The main contributions of our work are as follows:

1. In response to the lack of prior graphs in telemetry data, we propose a dynamic graph learning method to uncover the hidden initial dependency relationships between telemetry data variables. This method is integrated into a spatio-temporal feature extraction network.
2. We propose a novel graph-based spatio-temporal neural network model to effectively capture the complex spatial and temporal relationships within telemetry data, incorporating an attention mechanism to identify the significance of variables across different time periods.
3. We conduct extensive experiments on two publicly available spacecraft datasets, comparing our method with state-of-the-art baseline methods. The experimental results demonstrate that our method outperforms all the mentioned baseline methods, achieving an average F1 score of 0.97, which is 2.07% higher than the best performing baseline model, thus proving the superiority of our approach.

The rest of this paper is organized as follows: Section 2 presents the definition and description of the telemetry anomaly detection problem, along with the overall methodology. Section 3 details the model framework and the principles of the associated methods. Section 4 provides an explanation and analysis of the experimental results. Finally, Section 5 concludes this paper.

## 2. Problem Statement

Telemetry data are typically represented as a complex multivariate time series, consisting of data collected by multiple sensors over equal spatial and temporal intervals. In this work, we define a multivariate time series $x\in R^{N\times T}$, where *N* denotes the number of variables (i.e., the input features) and *T* represents the length of the time series. To enhance the feature representation of the training samples, a sliding window approach is applied to the raw telemetry data. This method divides the time series into multiple multivariate sub-sequences $W_{t}$, each with an equal window length τ. Specifically, each sub-sequence can be expressed as $W_{t}=\left\{X_{t-\tau +1},\ldots ,X_{t-1},X_{t}\right\}\in R^{N\times \tau }$, where $X_{t}=\left\{x_{t,1},\ldots ,x_{t,2},x_{t,N}\right\}\in R^{N}$ is the vector of all telemetry variables at time *t*. Here, τ denotes the size of the sliding window. After applying the sliding window, the processed input data become a set of sub-sequences $W=\{W_{1},W_{2},\ldots ,W_{n}\}$. The one-sided sliding window design ensures that only past data up to time *t* is included in $W_{t}$, maintaining causality and preventing data leakage from future observations. This is particularly critical for real-time anomaly detection tasks. To handle the initial timestamps (t<τ) where fewer than τ data points are available, we applied zero-padding to ensure that all windows are of consistent length. The goal of our approach is to take telemetry data *x*, preprocess them using the sliding window to generate *W*, and input them into the proposed STGLR model to compute the anomaly score for $X_{t}$. By comparing this score with a predefined threshold, the model determines whether the timestamp *t* corresponds to an anomaly and outputs a binary label $y_{t}\in \left\{0,1\right\}$.

**Figure 2:**
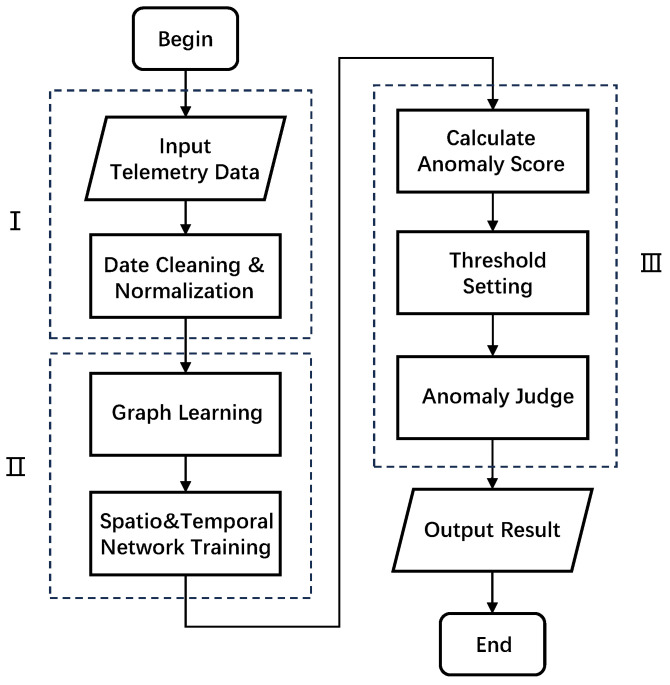
Part I is the data preprocessing process, part II is the spatio-temporal graph learning network training process, and part III is the anomaly detection process.

The overall framework of the proposed spacecraft anomaly detection method based on STGLR is shown in Figure 2. First, the telemetry data are preprocessed, which mainly includes data cleaning and normalization. Then, the preprocessed data are input into our STGLR model. Due to the imbalance between normal and abnormal data in the telemetry data, we train our model in an unsupervised manner. We train the network using normal data and then input the anomalous telemetry data into our trained model to validate its performance. We use normal data as the input for training the model to extract the temporal and spatial dependencies of the data, as well as to capture patterns in normal data. After training the model, we input the abnormal data, calculate the anomaly scores for all time points, set an appropriate threshold for anomaly detection, generate predicted anomaly labels, and compare them with the true labels to observe the performance of the model in anomaly detection.

## 3. Methodology

**Figure 3:**
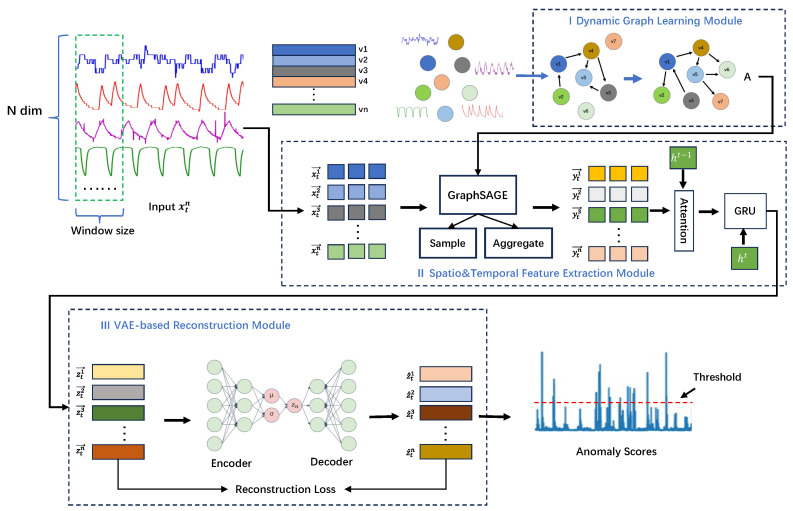
Overview of our proposed STGLR model architecture.

The architecture of the proposed STGLR model, as illustrated in Figure 3, consists of three primary components: (1) dynamic graph learning (DGL) module, which captures the latent relationships among telemetry data variables and constructs the graph structure; (2) spatio-temporal feature extraction module, which incorporates GraphSAGE to capture inter-variable relationships and utilizes a GRU model with an attention mechanism to extract temporal features and adaptively learn the importance of embedded features; and (3) variational autoencoder (VAE)-based reconstruction module, which reconstructs the input data and calculates anomaly scores for effective anomaly detection.

### 3.1. Dynamic Graph Learning Module

Spacecraft telemetry data lack a predefined graph structure. Many existing graph learning methods construct adjacency matrices by computing various distances or similarities between time series variables, such as Gaussian radial basis [32] and Pearson similarity [33]. Using fixed criteria to compute a static graph structure is neither comprehensive nor accurate. Because calculations are required at each time step, this can easily lead to long computation times for algorithms, especially when the time series is long and the number of variables is large. In order to better describe and capture the relationships between telemetry data variables, we designed a multivariate correlation dynamic graph learning module to adaptively learn and construct the graph of telemetry data. The graph between multivariate variables can be represented by G=(A,X), where *A* is the adjacency matrix, representing the topological structure of the graph, which can be characterized by a set of *N* nodes and a set of edges. $A_{ij}$ represents the (i,j) item and *X* is the node feature matrix, containing the attributes of each node, where the $i_{th}$ row $x_{i}\in R^{d}$ represents the *d* dimension feature vector of the node. We learn the graph and the entire model through a more direct optimization approach. As the model trains, the relationships between the nodes of the graph are continuously updated, and the constructed adjacency matrix *A* is dynamically updated accordingly, enabling continuous learning of the complex relationships between variables. Due to its ability to leverage GPU parallelization, the proposed DGL method demonstrates superior performance in large-scale data scenarios. The specific formula is as follows:

(1) $$\begin{array}{cc} \; & \left\{\begin{array}{c} \tilde{V^{1}}=\tanh\left(\delta V^{1}W_{gl}^{1}\right)\\ \tilde{V^{2}}=\tanh\left(\delta V^{2}W_{gl}^{2}\right)\\ A_{ij}=\mathrm{ReLU}\left(\tanh\left(\delta \tilde{V_{lj}^{1}}{\tilde{V_{lj}^{2}}}^{T}-\delta \tilde{V_{lj}^{2}}{\tilde{V_{lj}^{1}}}^{T}\right)\right) \end{array}\right. \end{array}$$

where $\tilde{V^{1}},\tilde{V^{2}}\in R^{N\times d}$ are represented by two neural networks with randomly initialized embedding matrices $V^{1},V^{2}$, each having N×d dimensions, where *N* is the number of sensors and *d* is the dimension of the network nodes, $W_{gl}^{1},W_{gl}^{2}\in R^{d\times d}$ are trainable parameter weight matrices with dimension d×d, and δ is a non-linear hyperparameter used to adjust the network activation saturation rate. In order to establish the directedness between variables, we calculate the difference between the node embedding matrices after two transformations are multiplied, and describe this directed relationship through two non-linear activation functions, ReLU and tanh, so as to normalize $A_{ij}$ between 0 and 1. In addition, in practical scenarios, not all sensors are correlated with each other. Referring to the GDN method [26], we introduce the Top-K algorithm, which only retains the top k nearest elements of each node in the adjacency matrix, meaning only the top K most relevant neighbor node relationships for each node are retained. This further reduces computational costs. The specific formula is as follows:

(2) $$\begin{array}{cc} \; & \left\{\begin{array}{c} \mathrm{TopK}=\mathrm{argmax}(A[i,:],k)\\ A_{ij}=1\left\{j\in \mathrm{TopK}\right\} \end{array}\right. \end{array}$$

where the argmax function is used to find the top *k* largest elements for each node in the adjacency matrix *A*. The value of *K* is determined based on the number of variables in the dataset and the effectiveness of anomaly detection. In subsequent ablation experiments, the experimental results of different values of *K* will be compared.

### 3.2. Spatio-Temporal Feature Extraction Module

In tasks involving multivariate time series, besides capturing temporal dependencies, considering relationships between variables is equally important. Thus, we propose a novel graph-based spatio-temporal neural network model.

#### 3.2.1. GraphSAGE

We employ the sampling and feature aggregation functions from the GraphSAGE network [23] to model spatial dependencies and accurately capture relationships between variables. Many GNN models aggregate node features in graphs using random walking strategy and require knowledge of the entire Laplacian matrix of the graph and information about neighboring nodes and thus can only be used for transductive tasks, such as a graph convolutional network (GCN). In contrast, GraphSAGE conducts operations only in the local neighborhood during each iteration, without requiring all neighboring nodes. Therefore, the GraphSAGE method can effectively generate embedding features for unknown nodes, exhibiting better versatility [23]. The GraphSAGE model generates node embeddings with the following procedures:

(1) Sample the domain nodes of the target node. Since the number of neighbors for each node varies, considering all neighbor nodes is inefficient. Instead, randomly sample several nodes first and then select neighboring nodes at the first, second, and Kth orders for sampling to improve overall computational efficiency. K represents the search depth from the target node.

(2) Aggregation of neighboring node features. The features of neighboring nodes of the target node $v_{i}$ sampled in the first step are aggregated *k* times using the Aggregate function to obtain embedding features for each node in the graph. Common types of Aggregate functions include MEAN and POOL. This paper employs the most commonly used MEAN function, with the specific formula as follows:

(3) $$\begin{array}{cc} \; & \left\{\begin{array}{c} h_{N(v)}^{\left(k\right)}={\mathrm{AGGREGATE}}^{\left(k\right)}\left(h_{N(v)}^{\left(k\right)}|u\in N\left(v\right)\right)\\ h_{N}^{\left(k\right)}=\sigma \left(W^{\left(k\right)}\cdot \mathrm{CONCAT}\left(h_{N(v)}^{\left(k\right)},h_{v}^{\left(k-1\right)}\right)\right) \end{array}\right. \end{array}$$

where the $h_{N(v)}^{\left(k\right)}$ is the embedded representation of all neighboring nodes of node *v* in the *k*-th layer, ${\mathrm{AGGREGATE}}^{\left(k\right)}$ is the aggregation function, CONCAT is the concatenation function, $W^{\left(k\right)}$ is the weight matrix of the *k*-th layer, and σ is the activation function.

#### 3.2.2. Attention-Based GRU Module

**Figure 4:**
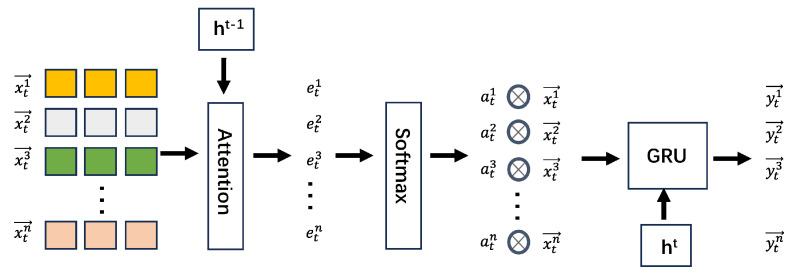
Attention-based GRU module.

We propose an Attention-based GRU module. This module adaptively selects the most relevant input features and learns the long-term temporal dependencies of the telemetry data. GRU [31], being simpler in design with fewer parameters compared to LSTM (Long Short-Term Memory) [34], leads to shorter training times and sometimes even better performance. Therefore, we employ the GRU to capture the temporal dependencies in telemetry data. In addition, an attention mechanism [35] is introduced to assess the importance of different variables in various time windows. As shown in Figure 4, before feeding embedded vectors into GRU, we combine the attention mechanism with the historical hidden states of GRU. Feature extraction is adaptively performed at each time step, followed by the computation of weighted coefficients, assigning different attention weights to different time steps. By incorporating the attention mechanism, the time feature extraction module can effectively capture long-term temporal dependencies in time series, adaptively selecting the most relevant input features, thus enhancing feature extraction capabilities.

The GRU model has an update and reset gate, which determine which information should be passed on and can retain information from a long time ago. The update gate helps the model decide how much past information to carry forward into the future, while the reset gate is used to decide how much past information to forget. The specific formulas are as follows:

(4) $$\begin{array}{cc} \; & \begin{array}{cc} \; & \left\{\begin{array}{c} Z_{t}=\sigma \left(W^{\left(z\right)}x_{t}+U^{\left(z\right)}h_{t-1}\right)\\ R_{t}=\sigma \left(W^{\left(r\right)}x_{t}+U^{\left(r\right)}h_{t-1}\right)\\ h_{t}^{\prime }=\tanh\left(Wx_{t}+R_{t}⊙Uh_{t-1}\right)\\ h_{t}=Z_{t}⊙h_{t-1}+\left(1-Z_{t}\right)⊙h_{t}^{\prime } \end{array}\right. \end{array} \end{array}$$

where $x_{t}$ is the input data, $W^{\left(z\right)}$ and $U^{\left(z\right)}$ are the weight matrices of the update gate, $W^{\left(r\right)}$ and $U^{\left(r\right)}$ are the weight matrices of the reset gate, $h_{t-1}$ is the historical state, and σ represents the sigmoid activation function. The current input, along with the historical memory state after passing through the reset gate and a non-linear layer, provides the new input information at the current time. Adding the result of the update gate gives the output $h_{t}$ of the GRU at the the current time.

We integrate an attention mechanism into the GRU model, enhancing its ability to extract relevant features. The process involves the following steps: first, the input sequence $x^{n}=\left\{x_{1}^{n},x_{2}^{n},\ldots ,x_{T}^{n}\right\}$ and the historical state $h_{t-1}$ are mapped to the attention space, where an attention weight matrix *W* and a bias term b are constructed. This is then passed through a linear layer to compute $m_{t}^{n}$. Subsequently, a softmax function is applied to ensure that all attention weights sum to 1. The attention weight $\alpha_{t}^{n}$ for each input feature is then calculated, establishing an attention-based feature extraction mechanism.

(5) $$m_{t}^{n}=\tanh\left(W\left[h_{t-1};x^{n}\right]+b\right)$$

(6) $$\alpha_{t}^{n}=\frac{\exp\left(m_{t}^{n}\right)}{\sum_{i=1}^{T}\exp\left(m_{t}^{i}\right)}$$

The attention-based input features, denoted as ${\vec{x_{t}}}^{\prime }$, are obtained by multiplying the attention weights with their corresponding input features. Then, by combining the historical state $h_{t-1}$ into the input of the GRU model to obtain the output $h_{t}$ at the current time.

(7) $${\vec{x_{t}}}^{\prime }=\left(\alpha_{t}^{1}x_{t}^{1},\alpha_{t}^{2}x_{t}^{2},\ldots ,\alpha_{t}^{n}x_{t}^{n}\right)$$

The proposed attention mechanism enables the temporal feature model to selectively focus on sequences with higher relevance, rather than extracting features uniformly from all input data. This further enhances the model’s capability to capture temporal dependencies within telemetry data.

### 3.3. VAE-Based Anomaly Detection Module

After capturing the spatial and temporal characteristics of telemetry data, the difference between normal and abnormal data needs to be measured in order to perform anomaly detection, so we also need to determine the reconstruction model. We choose the VAE [36] reconstruction model for learning the complex distribution of the data in the latent space, the VAE consists of an encoder–decoder structure. The encoder is able to represent the input $x_{t}$ as a downscaled latent representation $z_{t}$, which obeys some kind of conditional distribution $p_{\theta }\left(x_{t}|z_{t}\right)$, and then the decoder can reconstruct the $x_{t}$ by $z_{t}$ by updating the parameters of the model so that the reconstruction has the closest to the $x_{t}$ of the data distribution. It is difficult to reconstruct $x_{t}$ from $z_{t}$, and the posterior distribution $q_{\varphi }\left(z_{t}|x_{t}\right)$ needs to be computed. The VAE transforms the Bayesian problem into an optimization problem by approximating the posterior distribution $q_{\varphi }\left(z_{t}|x_{t}\right)$ through variational inference and minimizing the KL scatter.

(8) $$\begin{array}{cc} \; & \left\{\begin{array}{c} p_{\theta }\left(z_{t},x_{t}\right)=p_{\theta }\left(z_{t}\right)p_{\theta }\left(x_{t}|z_{t}\right)\;\\ q^{*}={\mathrm{argmin}}_{q_{\varphi }\left(z_{t}\right)\in Q}KL\left(q_{\varphi }\left(z_{t}\right)\left|\right|p_{\theta }\left(z_{t}|x_{t}\right)\right) \end{array}\right. \end{array}$$

(9) $$\begin{array}{cc} ELBO & =E_{q_{\varphi }\left(z_{t}|x_{t}\right)}\left[\log\frac{p_{\theta }\left(x_{t}|z_{t}\right)}{q_{\varphi }\left(z_{t}|x_{t}\right)}\right]\\ \; & =E_{q_{\varphi }\left(z_{t}|x_{t}\right)}\left[\log p_{\theta }\left(x_{t}|z_{t}\right)\right]\\ \; & -KL\left[q_{\varphi }\left(z_{t}|x_{t}\right)\left|\right|p_{\theta }\left(z_{t}\right)\right] \end{array}$$

We use the commonly used stochastic gradient variational Bayesian (SGVB) method to solve the ELBO (Evidence Lower Bound) with the following formulae, where φ and θ are the training parameters of the generative and inference networks, respectively. Reconstruction loss generally consists of two parts, the first part is reconstruction loss (reconstruction loss). Reconstruction loss is used to measure the difference between reconstructed data and original data, which can be approximated by sampling multiple $z_{t}$ from the prior distribution $p_{\theta }\left(x_{t}|z_{t}\right)$, and if the data are assumed to be Gaussian with fixed variance, the objective function obtained after the maximum likelihood estimation is equivalent to the MSE. The second component is the regularization term of the latent space, which is usually measured using the KL scattering loss ( KL divergence loss) to measure the difference between the distribution of the latent variable and the standard normal distribution.

(10) $$\begin{array}{cc} L_{re} & =L_{\mathrm{Recon}\;}+L_{KL}\\ \; & =∥x-\hat{x}{∥}^{2}+KL\left[N\left(\mu_{x},\sigma_{x}\right),N\left(0,1\right)\right] \end{array}$$

The reconstruction model is trained to learn the underlying spatial distribution of the normal data, so if the input data $x^{t}$ has anomaly at time t, then the reconstructed data ${\hat{x}}_{i}^{t}$ has a high probability of being significantly different from the original data, so we choose the reconstruction error of the VAE to compute the anomaly score for anomaly detection. To prevent the deviation of a particular telemetry value from differing excessively compared to other telemetry values, we normalize the anomaly scores of each telemetry value.

(11) $$\;\mathrm{Anomaly}\;\mathrm{Scores}\;=\frac{1}{S}\sum_{i=1}^{S}\left|x_{i}^{t}-{\hat{x}}_{i}^{t}\right|$$

If the anomaly score is low, it means that the input data $x^{t}$ can be reconstructed better, indicating that $x^{t}$ comprises normal data. On the other hand, if the anomaly score is high, it means that the data distribution is different from the learned normal data distribution, and it is likely that there is an anomaly. By setting a suitable threshold, better detection can be achieved. We adopt the Peak Over Threshold (POT) [16] algorithm to calculate the anomaly threshold on the validation set. POT is a threshold selection method based on extreme value theory, which fits the tail of the probability distribution using a parameterized Generalized Pareto Distribution (GPD). The detection rule is that if the anomaly score at a given timestamp exceeds the computed threshold, it is labeled as “abnormal”; otherwise, it is labeled as “normal”.

The complete STGLR process is shown in Algorithm 1, where τ is the size of the sliding window, *K* is the value of K in the Top-K method of the dynamic graph learning module, *N* represents the dimensionality of the input data, and *B* is the size of the model training batch. The process begins with windowing the input data, learning the dynamic relationships between variables using the DGL model, obtaining the adjacency matrix $\hat{A}$, and obtaining embedded vectors $\vec{y_{n}^{t}}$ through GraphSAGE. Then, it involves inputting node embedding into the GRU model combined with attention mechanism to compute the weights of different time variables and obtaining $\vec{z_{n}^{t}}$. Next, the VAE model is used to reconstruct $\vec{z_{n}^{t}}$ to obtain $\hat{z_{t}^{n}}$. Finally, the reconstruction loss is calculated and the parameters of the entire model are updated based on the loss $L_{re}$.
**Algorithm 1** General framework of STGLR**Require:** Telemetry dataset *O*,epoch,τ,*K*,*n*,*B*,len;**Ensure:** Reconstruction loss;  1: **for** *i* = 1 to epoch **do**  2:       **for** *t* = τ to len **do**  3:             Sample $\vec{x_{n}^{t}}\in R^{B\times N\times \tau }$ from *O*  4:             $\hat{A}\leftarrow \mathrm{DGL}\left(X,N,K\right)$ using  5:             $\vec{y_{n}^{t}}\leftarrow \mathrm{GraphSAGE}\left(\vec{x_{n}^{t}},\hat{A}\right)$ using  6:             $\vec{z_{n}^{t}}\leftarrow \mathrm{Att}-\mathrm{GRU}\left(\vec{y_{n}^{t}}\right)$ using  7:             $\hat{z_{t}^{n}}\leftarrow \mathrm{VAE}\left(\vec{z_{n}^{t}}\right)$ using  8:             Calculate $L_{re}$ using  9:             Back propagation $L_{re}$ update model parameters10:       **end for**11:       i←i+112: **end for**

## 4. Experiments

In this section, we conducted numerous experiments to demonstrate the effectiveness of the STGLR method. We first introduced two widely used public datasets. We evaluated STGLR on these two datasets and showed that STGLR outperforms current anomaly detection methods significantly. Next, we conducted an ablation study on the key components of the proposed model. Finally, we evaluated the effectiveness of STGLR through a case study.

### 4.1. Datasets and Evaluation Metrics

We conducted experiments on two publicly available NASA datasets: the Soil Moisture Active Passive (SMAP) spacecraft and the Mars Science Laboratory (MSL) rover [12]. They contain anomalous data and corresponding labels, each with multiple channels, with dimensions of 55 and 25, respectively. Furthermore, they are divided into training and testing sets. Only the testing set contains anomalous data, which is used for our unsupervised algorithm. Detailed information about the datasets is presented in Table 1.

We use the most widely recognized and authoritative metrics in the anomaly detection field for algorithm evaluation, namely precision, recall, and F1 score. Here, TP, FP, and FN represent the numbers of true positives, false positives, and false negatives, respectively. Additionally, we adopt the widely used point adjustment strategy [16] to evaluate the proposed method: if our method detects an anomaly at a certain time point within a continuous anomaly, it is considered that all anomalies in that continuous segment have been correctly detected. This strategy is reasonable since anomalies annotated by experts in real data are often continuous time periods. Therefore, detecting an anomaly at a specific time point implies the successful detection of anomalies within that time segment.

(12) $$Precision=\frac{TP}{TP+FP}$$

(13) $$Recall=\frac{TP}{TP+FN}$$

(14) $$F1-score=\frac{2\times Precision\times Recall}{Precision+Recall}$$

### 4.2. Experimental Settings

We implemented our proposed method on PyTorch version 2.0.1 with CUDA 11.8. The overall experiments were conducted on an Intel Core i9-13900K @3.00 GHz processor and an NVIDIA GeForce RTX 4090 graphics card. The sliding window size for MSL is set to 20, with a learning rate of 0.01. The sliding window size for SMAP is set to 100, with a learning rate of 0.001. To prevent overfitting, dropout is set to 0.2, and the Adam optimizer is used for training. Batch sizes of 240 and 10 epochs are used for training. The embedding output for GraphSAGE is 5, and the embedding dimension for the VAE is set to 100.

### 4.3. Baselines

In order to demonstrate the effectiveness of our proposed method, we will compare it with other advanced anomaly detection methods, including those based on reconstruction and prediction models.

LSTM-NDT [12]: The MSL dataset and SMAP dataset are disclosed in this paper, where the authors use LSTM-based prediction models for detection and propose a non-parametric threshold calculation method.

LSTM-VAE [37]: A reconstruction-based anomaly detection model that introduces LSTM for extracting temporal features from multidimensional signals to achieve improved performance.

OmniAnomaly [16]: Utilizes methods such as random variable connections and planar normalized flows to learn representations of multivariate time series and explains detected anomalies by calculating reconstruction probabilities through VAE.

MAD-GAN [38]: Combines prediction and reconstruction methods while employing two GANs to learn the temporal and feature correlations of multivariate time series for improved temporal feature representation.

MTAD-GAN [25]: Combines prediction and reconstruction methods and uses two GANs to learn the correlation of multivariate time series and feature dimensions for better representation of time-series features.

GDN [26]: GDN combines structural learning methods with graph neural networks and introduces attention mechanisms to learn the complex relationships and biases among variables for accurately detecting anomalies in time series data.

InterFusion [39]: An unsupervised anomaly detection method based on hierarchical VAE that can model the spatio-temporal dependencies of multivariate time series.

DyGraphAD [28]: DyGraphAD detects anomalies by evolving graphs based on the deviation from abnormal to normal states in multivariate time series, leveraging the dynamic changes in graphs.

Considering the diversity of variables (features) and time series trends in different datasets, we search for optimized combinations of key hyperparameters for each dataset to achieve the best performance. These hyperparameter settings are shown in Table 2. For the learning rate and maximum epochs, we refer to OmniAnomaly [16] and further fine-tune based on the number of parameters in our model. We conducted ten random experiments using different random seeds for our model, with the final results reported as the average of all metrics. For baseline models with publicly available source code, we used the parameter settings specified in their respective papers to ensure a fair comparison. Each baseline model was also evaluated using ten random experiments, and the average values were reported as the final evaluation results. The results of the InterFusion method are reported in [39].

### 4.4. Results and Comparisons

The experimental results of our method and all baseline methods on two public datasets are shown in Table 3. It can be observed that our proposed method demonstrates superior performance. Although certain baseline methods achieved excellent results in a single metric, STGLR demonstrates a more balanced precision and recall, with the most representative F1 scores reaching 0.978 and 0.972, respectively, averaging above 0.97. For the SMAP dataset, STGLR performs 14.7%, 10.5%, and 2.9% better in terms of the F1 score compared to LSTM-VAE, Omnianomaly, and DyGraphAD, respectively. STGLR also achieves competitive performance on precision and recall metrics.

In summary, we draw the following conclusions: The STGLR method demonstrates superior effectiveness compared to LSTM-NDT, LSTM-VAE, and OmniAnomaly, as these models only capture the temporal dependencies in time series data, neglecting the complex interrelationships between variables. While MAD-GAN and InterFusion account for both temporal and spatial dependencies, they input all variables into the model simultaneously, failing to explicitly model the relationships between them. Consequently, their anomaly detection performance is suboptimal. This highlights the importance of considering inter-variable relationships and feature extraction. Although MTAD-GAN introduces graph neural networks to capture spatial dependencies, enhancing anomaly detection performance, its static graph structure is overly simplistic. Constructing node relationships using one-dimensional convolutions does not accurately reflect real-world scenarios. GDN represents initial relationships between variables by calculating their similarity; however, this approach has high time and space complexity, and similarity measures cannot fully capture the correlations between variables, leading to less-than-ideal detection performance. DyGraphAD detects anomalies by identifying temporal correlation differences between normal and anomalous states in time series data. However, this model, along with others, places greater emphasis on temporal features, resulting in biased learned variable relationships. Compared to the strongest baseline, DyGraphAD, our method achieves an average performance improvement of 2.1%, marking a significant performance enhancement. The STGLR method outperforms these baseline models on the MSL and SMAP datasets due to its graph learning approach, which more accurately represents the initial relationships between variables, effectively captures correlations between variables in telemetry data, and learns both temporal and spatial dependencies, thereby achieving superior anomaly detection results.

### 4.5. Ablation Studies

To validate the effectiveness of each module in STGLR, we conducted ablation experiments by excluding or replacing several key components. We demonstrate the effectiveness of the main modules in STGLR through comparisons: (1) dynamic graph learning (DGL), (2) GraphSAGE, and (3) GRU&Att.

The model was reconstructed to create the following types of variants:

(1) w/o DGL: This variant disables the dynamic graph construction module and replaces it with a method that randomly generates adjacency matrices.
(2) w/o GraphSAGE: This variant disables GraphSAGE and does not capture correlations between variables, processing the time window data directly as input.
(3) w/o GRU&Att: This variant disables the GRU&Att module and inputs the node features extracted by the GNN directly into the reconstruction model.
(4) w/o Attention: This variant disables the Attention module, i.e., only the GRU model is used.
(5) w/o RNN: Replaces GRU&Att with the RNN model.

The experimental setup for these variant models was consistent with that of the full model, with the only differences being the removal or replacement of specific components. The results in Table 4 demonstrate that our original model outperforms the variants across all metrics and datasets.

Impact of DGL (w/o DGL): To assess the effectiveness of dynamic graph learning, we replaced the dynamic graph construction method. This resulted in a noticeable decline in detection performance, with the F1 score decreasing by 4.5% on the MSL dataset and 4.4% on the SMAP dataset. The results indicate that our dynamic graph construction method effectively captures the initial relationships between multiple variables.

Impact of GraphSAGE (w/o GraphSAGE): To examine the importance of the graph neural network, we disabled the GraphSAGE network, resulting in a significant drop in detection performance. The F1 score decreased by 27% on the SMAP dataset and by 6.7% on the MSL dataset, with the latter experiencing the most pronounced decline. These results underscore the critical importance of considering complex inter-variable relationships when performing multivariate time series feature extraction.

Impact of the Temporal Feature Extraction Module (w/o GRU&Att, w/o RNN, w/o Attention): As shown in the table, the removal of the temporal feature extraction module leads to a decrease in anomaly detection performance, with the average F1 score dropping by 2.1%. This result indicates the effectiveness of the module. When replacing GRU with RNN, performance declines, as reflected by a 0.9% reduction in the average F1 score. This is because RNN struggles to capture long-term dependencies in time series data. Additionally, using only the GRU model without integrating the attention mechanism also results in performance degradation, with the F1 score on the SMAP dataset dropping by 0.6%. This demonstrates the effectiveness of incorporating the attention mechanism in temporal feature extraction.

The results of ablation experiments indicate that removing any component of the model leads to a decrease in anomaly detection performance, demonstrating the importance and effectiveness of each component in STGLR. The key components are the GraphSAGE graph neural network and the dynamic graph learning network, which demonstrate the criticality of both temporal and spatial features in multivariate time series and the importance of capturing initial relationships in the data. Furthermore, our GRU model combined with attention mechanisms achieved significant performance gains, validating the superiority of our approach.

### 4.6. Parameter Analyses

This section investigates the sensitivity of several key hyperparameters in the STGLR model, including window size, the value of k for nearest neighbors in the DGL module, batch size, and the selection of the GNN model, to evaluate their impact on the performance of the STGLR method. The experimental setup remains consistent with previous experiments, and the following analysis is derived from the results.

**Figure 5:**
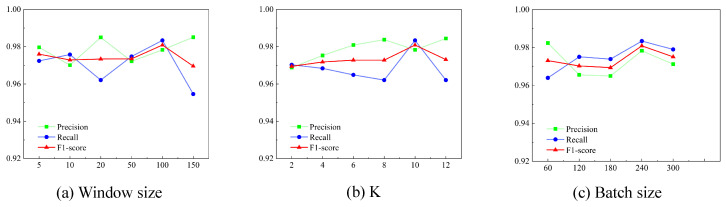
Experimental results of the SMAP dataset tuning each parameter.

**Figure 6:**
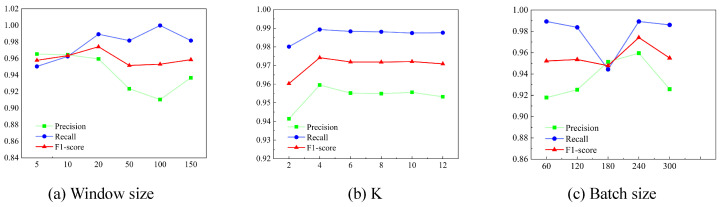
Experimental results of the MSL dataset tuning each parameter.

Window size. Selecting an appropriate window size is crucial for improving anomaly detection performance. Appropriately increasing the window size can improve precision, recall, and the F1 score. However, after a certain threshold, further increasing the window size leads to a decline in effectiveness. As shown in Figure 5a and Figure 6a, the performance evaluation indicates that for the SMAP dataset, a window size of 100 yielded the highest F1 score, while for the MSL dataset, the optimal window size was 20. This decrease may be due to the introduction of post-detection anomalies, as excessively large window sizes increase the likelihood of model errors.

Batch size. Within a reasonable range, increasing the batch size appropriately can enhance model performance, accelerate computation, and reduce the impact of noise on model training. As shown in Figure 5b and Figure 6b, an F1 score peak is observed for both datasets when the batch size is around 240. However, excessively large or small batch sizes can lead to suboptimal results, and the choice of batch size should also account for the memory capacity of the training hardware.

The size of the k in the Top-K method. The k-value plays a crucial role in determining the effectiveness and efficiency of the dynamic graph learning method. Employing the Top-K method and selecting an appropriate k-value can significantly improve training efficiency. A k-value that is too small may result in the loss of critical data, while a k-value that is too large can retain excessive noise, leading to higher computational costs. As shown in Figure 5c and Figure 6c, the effectiveness decreases when the k-value is either too small or too large. The optimal k-value is 10 for the SMAP dataset and 4 for the MSL dataset.

Selection of GNN models. Several classic GNN models were mainly compared, including GCN [40], GAN [22], and ChebyNet [41]. GraphSAGE was used for comparison with other models, and as shown in Table 5, it delivered the best performance. Its average F1 score exceeded that of the widely used GAN by 5.69%. This is attributed to GraphSAGE’s inductive reasoning, which allows it to handle unseen nodes and exhibit stronger generalization capabilities [23].

### 4.7. Case Studies

To provide a clear understanding of the effectiveness of our anomaly detection model, we conducted case studies and visualized key anomalous data to validate critical aspects of the model design. Although both datasets contain numerous variables, only a few show significant correlations in the telemetry data. We selected five representative variables from the SMAP and MSL datasets, respectively, for detailed analysis and plotted them in a single figure. The vertical axis denotes the variables, which are represented by symbols with subscripts due to confidentiality regarding the specific names of the telemetry parameters. The horizontal axis indicates the length of the variables, with 300 sampling points being chosen. The figure includes both some typical anomalies and certain types of anomalies that are difficult to detect.

**Figure 7:**
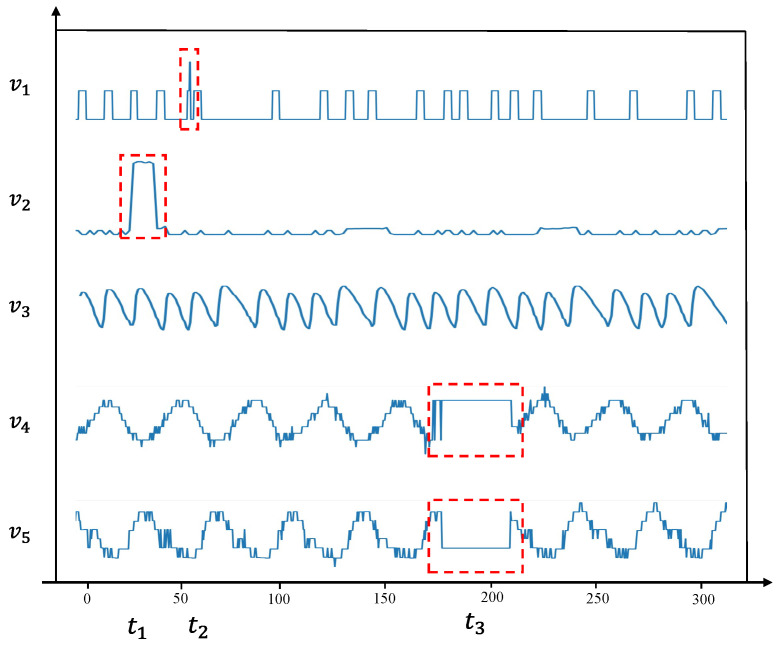
A case study of five variables in the SMAP dataset, which includes typical point anomalies and collective anomalies.

The red dashed boxes highlight the anomalies detected by our model, which align with the actual anomalous regions. As shown in Figure 7, variables v1 and v2 display distinct anomalies at time points t1 and t2, respectively, with values in the anomalous regions differing significantly from nearby normal values. The anomaly in v1 is a typical point anomaly, characterized by noticeable changes at only a few time points. In contrast, v2 exhibits a collective anomaly, occurring over a continuous period. Such anomalies, with large value deviations, can be detected relatively easily by both the proposed method and other anomaly detection approaches. Variable v3 shows no anomalies and demonstrates a regular periodic pattern. Variables v4 and v5 follow similar trends and display a strong correlation. At time t3, both variables suddenly exhibit fixed values, diverging from their original trends, indicating a multivariate anomaly, despite the values remaining within a normal range. Traditional anomaly detection methods, such as threshold-based approaches, struggle to detect this type of anomaly. However, STGLR effectively identifies it by capturing the temporal dependencies in the telemetry data, including periodic features. Additionally, our use of GraphSAGE to model the relationships between variables enhances the detection of anomalies in highly correlated variables.

**Figure 8:**
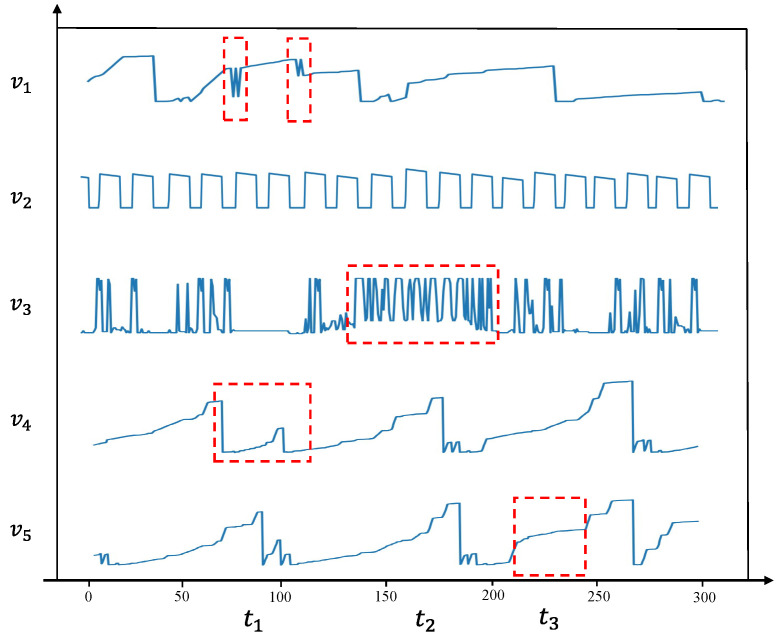
A case study of five variables in the MSL dataset, which includes challenging-to-detect anomaly types and multivariate anomalies.

As shown in Figure 8, variable v1 exhibits a typical point anomaly at time t1, showing a significant change in value. The time series for variable v2 follows a largely cyclic pattern, with no anomalies detected. Variable v3 experiences a significant acceleration in data fluctuation frequency at time t2. Although these values remain within the normal range and no other variables show significant changes at that time, such anomalies are typically difficult to detect. However, our model successfully identified this anomaly. This success is attributed to the attention mechanism integrated into the GRU model, which assigns different weights to variables based on their temporal dependencies. As a result, the model focuses more on variables with notable temporal feature changes, enabling it to detect this type of anomaly. Variables v4 and v5 exhibit very similar trends, indicating a strong correlation between them. Upon examining their graphs, it becomes clear that both variables display slight trend changes at times t1 and t3. Specifically, v4 shows an early decline at t1, while v5 shows a change in rate from fast to slow at t3. Although these changes are small, making it difficult to definitively identify them as anomalies, our model successfully detected anomalies at both points. This is because the model accounts for the correlations between strongly related variables. When one variable’s trend diverges from that of other highly correlated variables, the likelihood of detecting an anomaly at that point increases.

## 5. Conclusions

This paper proposes a novel anomaly detection method for spacecraft telemetry data, referred to as STGLR. Specifically, we integrate graph neural networks to capture the complex spatial dependencies in telemetry data. Compared to methods that do not explicitly model variable relationships, such as LSTM-VAE [37] and OmniAnomaly [16], our approach achieves higher precision and recall. To address the lack of an initial graph structure in telemetry data, we design a dynamic graph learning (DGL) method to capture the initial relationships between variables and generate the corresponding adjacency matrix. Compared to the correlation-based graph construction method used in GDN [26], the anomaly detection performance of DGL is superior, as demonstrated by the baseline comparison results in Table 3. The features of the variables are learned using the inductive learning approach of GraphSAGE. In comparison to other graph neural network models such as GCN [40] and GAN [22], GraphSAGE’s random sampling demonstrates better generalisability and more effective feature extraction, as shown in Table 5. The long-term temporal dependencies in telemetry data are extracted using the encoder component of GRU. Additionally, we introduce an attention mechanism to adaptively assign weights to variables over different time intervals, enabling the selection of critical features. The performance improvement is illustrated in Table 4. Finally, a reconstruction model based on VAE is employed to reconstruct feature vectors and learn the latent representations of normal data. According to the case study results in Section 4.7, the proposed STGLR method effectively detects various types of anomalies.

To validate the effectiveness and superiority of the proposed anomaly detection method, experiments were conducted on two publicly available spacecraft telemetry datasets, MSL and SMAP. The experimental results demonstrate that the proposed STGLR method achieves the best overall performance compared to other state-of-the-art anomaly detection techniques. Future work will focus on reducing training time, exploring online training methods, and applying this approach to a broader range of spacecraft telemetry data.

## Figures and Tables

**Table 1:** Details of data.

Dataset	Variable Number	Training Set Size	Testing Set Size	Anomaly Ratio (%)
MSL	55	58,317	73,729	10.72
SMAP	25	135,183	427,617	13.13

**Table 2:** Parameters’ setting.

Parameters	MSL	SMAP
DGL neighbor size k	4	10
DGL node dimension	128	128
Number of GraphSAGE Layers	2	2
GraphSAGE hidden dimension	16	16
VAE hidden dimension	100	100
Batch size	240	240
Learning rate	0.01	0.001
Dropout	0.2	0.2
Window size	20	100
Optimizer	Adam	Adam
Loss Function	MSE	MSE

**Table 3:** Quantitative results of STGLR (ours) across two real telemetry datasets. Herein, P, R, and F1 signify the precision, recall, and f1-score, respectively. The R-model and P-model signify the reconstruction and prediction models, respectively. STGLR performs best on F1 scores, indicated in bold.

		SMAP			MSL		
	**Method**	**P**	**R**	**F1**	**P**	**R**	**F1**
R-model	LSTM-VAE [37]	0.716	0.988	0.830	0.855	0.799	0.826
	MAD-GAN [38]	0.805	0.821	0.813	0.852	0.899	0.875
	OmniAnomaly [16]	0.813	0.942	0.873	0.927	0.850	0.887
	InterFusion [39]	0.898	0.885	0.891	0.813	0.927	0.866
P-model	LSTM-NDT [12]	0.846	0.910	0.876	0.969	0.693	0.808
	MTAD-GAN [25]	0.891	0.912	0.901	0.875	0.944	0.908
	GDN [26]	0.893	0.887	0.890	0.914	0.861	0.887
	DyGraphAD [28]	0.934	0.966	0.949	0.970	0.949	0.959
	**Ours(STGLR)**	0.978	0.978	**0.978**	0.951	0.995	**0.972**

**Table 4:** Ablation studies. The complete model performs best in F1 score, indicated in bold.

	SMAP			MSL		
**Method**	**P**	**R**	**F1**	**P**	**R**	**F1**
w/o DGL	0.922	0.945	0.933	0.922	0.933	0.928
w/o GraphSAGE	0.935	0.569	0.707	0.850	0.967	0.905
w/o GRU & Att	0.957	0.976	0.966	0.950	0.934	0.942
w/o RNN	0.959	0.983	0.971	0.965	0.958	0.962
w/o Attention	0.981	0.965	0.973	0.950	0.981	0.965
**Ours**	0.978	0.978	**0.978**	0.951	0.995	**0.972**

**Table 5:** GNN model results. The GraphSGAE we use performs the best on all metrics, indicated in bold.

	SMAP			MSL		
**GNN Module**	**P**	**R**	**F1**	**P**	**R**	**F1**
GCN	0.683	0.868	0.764	0.916	0.995	0.954
GAN	0.961	0.809	0.879	0.919	0.864	0.958
ChebyNet	0.734	0.845	0.785	0.948	0.956	0.952
**Ours**	**0.978**	**0.978**	**0.978**	**0.951**	**0.995**	**0.972**

## Data Availability

The datasets used in this study are available in the telemanom repository at https://github.com/khundman/telemanom (accessed on 1 March 2024).

## Abbreviations

The following abbreviations are used in this manuscript:

STGLR	Space–time graph learning and reconstruction
GNN	Graph neural network
GraphSAGE	Graph sample and aggregation
GRU	Gated recurrent unit
DTW	Dynamic time warping
DGL	Dynamic graph learning
VAE	Variational autoencoder
GCN	Graph convolutional network
